# Aptamer-Based Platforms for Human Aging Biomarkers: Multiplexed Proteomics, Biosensors and Translational Perspectives

**DOI:** 10.3390/ijms27177580

**Published:** 2026-08-24

**Authors:** Zulfiya Guvatova, Anastasiya Kobelyatskaya, Alexander Gorbunov, Elena Pudova, Alexey Moskalev

**Affiliations:** 1 Petrovsky National Research Center of Surgery, Institute of Biology of Ageing and Healthy Longevity Medicine with Clinic of Predictive Medicine, 119435 Moscow, Russia; guvatova_z@eimb.ru (Z.G.); kaa.chel@mail.ru (A.K.); pudova_elena@inbox.ru (E.P.); 2 Engelhardt Institute of Molecular Biology, 119991 Moscow, Russia; 3 Pharmacology Department of the Institute of Digital Biodesign and Artificial Intelligence in Medicine, Sechenov University, 119435 Moscow, Russia; gorbunov_a_a@staff.sechenov.ru

**Keywords:** aptamers, SELEX, SOMAmer, SomaScan, aging biomarkers, proteomic aging clock, aptasensors, senescence

## Abstract

Aptamer-based multiplexed proteomic platforms, especially the SOMAmer-based SomaScan assay, are widely used for large-scale discovery of circulating biomarkers relevant to human aging. This review summarizes 42 original research articles published from 2020 through 2026 in which aptamers or aptamer-derived biosensors were used to characterize aging-related biomarkers in human samples or clinically relevant human-disease contexts. The eligible literature falls into several thematic areas: whole-plasma and organ-specific proteomic aging clocks; inflammaging and senescence-associated secretory phenotype (SASP) markers; cardiovascular, metabolic, renal, hepatic, musculoskeletal and neurodegenerative biomarker panels; and aptasensor platforms for detection of individual analytes. Only a small number of studies have compared aptamer- and antibody-based platforms in the same specimens; we tabulate these and show that median between-platform agreement is low to moderate, which constrains the pooling of findings across technologies. We also make explicit an interpretive point that is usually left implicit: because proteomic clocks are trained against chronological age, their correlation with chronological age measures fit to the training target rather than biological validity, and the informative quantity is the residual age gap. In the reviewed literature, SomaScan-based studies are concentrated in cardiovascular, neurodegenerative, frailty, and proteomic aging-clock research, whereas de novo SELEX campaigns targeting aging-specific epitopes and longitudinal human validation of wearable aptasensors were not identified. The main barriers to translation are cross-platform discordance, limited replication across ancestries, under-reported pre-analytical variability, cost, and the research-use-only status of most assays.

## 1. Introduction

### 1.1. Biomarkers of Aging: Concept and Classification

Chronological age is an imperfect predictor of individual health trajectories: people of the same calendar age differ substantially in disease risk, physical function, and remaining lifespan [1,2]. This discrepancy has motivated the search for “biological age” estimators—quantitative biomarkers or composite scores that track the cumulative burden of molecular and cellular damage more closely than birth date alone [2,3]. Candidate biomarkers are drawn from several classes of measurement, each interrogating a different level of biological regulation: DNA methylation at defined CpG sites, from which epigenetic clocks are computed [4,5,6,7]; age-associated changes in the abundance of blood transcripts [8]; circulating metabolites [9]; and plasma or serum proteins [10,11]. Among these, the plasma and serum proteome occupies a distinctive position: proteins are the direct effectors of most physiological processes, they integrate signals from essentially every organ system, and they are readily accessible through minimally invasive blood sampling [12,13,14]. Proteomic biomarkers of aging are therefore of particular interest both for mechanistic research into the hallmarks of aging [1] and for practical risk stratification in clinical and translational geroscience. Whichever class of measurement is used, a candidate biomarker of aging is not established by its correlation with chronological age alone: current consensus criteria additionally require a demonstrated association with adverse health outcomes, responsiveness to intervention and adequate reliability at the individual rather than the population level [15]. This requirement determines how the evidence is assessed throughout the present review.

### 1.2. Aptamers as Molecular Recognition Elements

Aptamers are short single-stranded DNA or RNA oligonucleotides that fold into defined three-dimensional structures and bind a target molecule with high affinity and specificity. They are obtained by iterative rounds of in vitro selection and amplification, a procedure known as Systematic Evolution of Ligands by EXponential enrichment (SELEX), introduced independently by two groups in 1990 [16,17] and since developed into a large family of variants [18].

The reagents used in the studies reviewed here belong to several chemically distinct classes, and it is worth distinguishing them because they are not interchangeable. Unmodified DNA aptamers are inexpensive, chemically robust, and simple to immobilize on an electrode, and they account for most of the biosensor work discussed in Section 2.3 and Section 5. RNA aptamers offer richer secondary structure but require 2′-fluoro or 2′-*O*-methyl pyrimidine substitution to resist serum nucleases; pegaptanib, the first aptamer approved for human use, is a modified, PEGylated RNA aptamer [19,20]. Mirror-image L-ribose analogs (Spiegelmers) achieve nuclease resistance through stereochemistry rather than substitution [21]. Slow off-rate modified aptamers (SOMAmers) constitute a fourth class and are the reagents on which the SomaScan assay is built [22].

**Figure 1:**
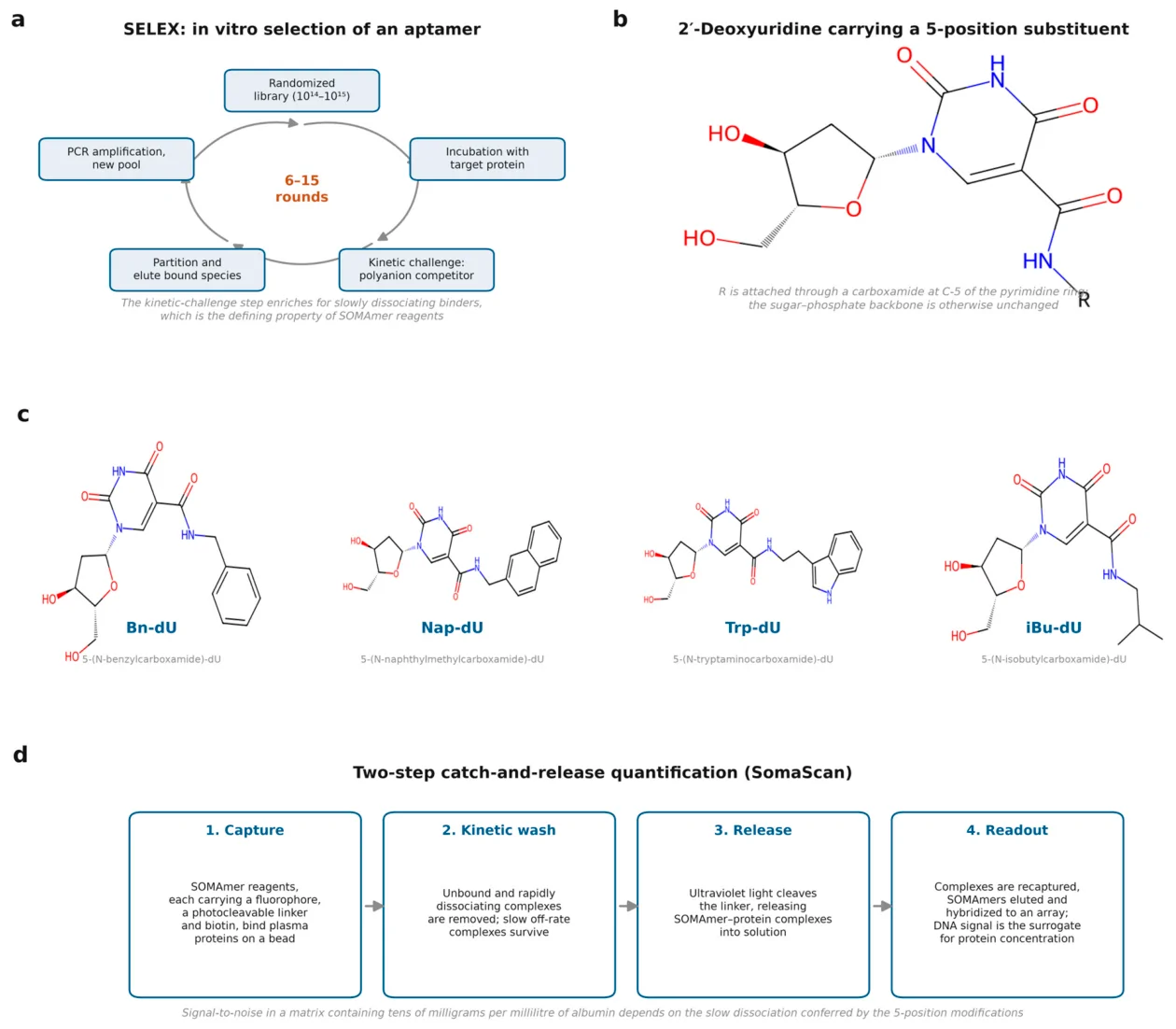
Chemistry and selection of SOMAmer reagents. (**a**) The SELEX cycle: a randomized oligonucleotide library is incubated with the target protein, bound species are partitioned from unbound, and the recovered pool is amplified and used as the input to the next round; SOMAmer selection additionally exploits a kinetic-challenge step that enriches for slowly dissociating binders. (**b**) 2′-Deoxyuridine carries a hydrophobic substituent at the 5-position of the pyrimidine ring (R). (**c**) Representative 5-position modifications used in SOMAmer libraries, which reproduce the aromatic and aliphatic side chains enriched in antibody paratopes. (**d**) Schematic of the two-step catch-and-release SomaScan procedure, in which slow dissociation of the modified aptamer–protein complex allows unbound and rapidly dissociating species to be washed away before the surviving complexes are quantified through their DNA tag.

SOMAmers carry hydrophobic substituents at the 5-position of deoxyuridine (Figure 1). These groups mimic the hydrophobic and aromatic amino-acid side chains that dominate antibody paratopes, and their introduction into the selection library substantially increased the proportion of protein targets for which high-affinity ligands could be obtained relative to unmodified DNA [22,23,24]. Co-crystal structures show that the modified nucleobases do more than add bulk: they form dedicated hydrophobic contacts and pack against shallow protein surfaces, producing interfaces that resemble antibody–antigen rather than classical nucleic-acid–protein complexes [25,26]. The resulting complexes dissociate slowly—the property from which the reagents take their name—and it is this slow off-rate that permits the two-step catch-and-release wash on which the assay depends, and that keeps signal-to-noise acceptable in a matrix as protein-dense as plasma [22,24].

At the same time, SOMAmers retain the advantages of nucleic-acid chemistry: animal-free in vitro production, batch-to-batch consistency, chemical stability, ease of multiplexing on a single array, and straightforward quantitative readout by DNA microarray or sequencing [27]. These properties underlie the scalability that has made the platform attractive for aging cohorts, and the supporting performance data are published rather than assumed. Assay-variability studies have characterized intra- and inter-plate coefficients of variation across control samples for successive panel versions [28,29]. At cohort scale, single-laboratory campaigns have profiled several thousand participants at once, as in the AGES-Reykjavik serum survey [30] and the INTERVAL plasma survey [31]. The effect of long-term specimen storage has also been examined directly, with SOMAmer signals in sera cryopreserved for extended periods remaining usable for proteomic analysis, although a subset of reagents showed storage-dependent drift [32].

Cross-laboratory comparability, which the above does not establish, is a weaker point for every platform in this field. SomaScan is run as a centralized service by a single provider, which removes site-to-site operator variation but replaces it with dependence on one vendor’s lot and version control; Olink is distributed across certified laboratories with a bridging-sample normalization scheme; mass spectrometry is the most decentralized of the three. None is currently supported by a certified reference material or an external quality-assessment scheme of the kind routine in clinical chemistry, and reported harmonization has instead relied on ad hoc bridging samples and restricting analyses to reagents shared between panel versions [29,33,34]. We return to this in Section 7 and Section 9.

This review concentrates on SOMAmer-based measurements not because the other aptamer chemistries are less interesting, but because of how the aging literature is actually distributed. Of the 42 eligible articles, 33 used the SomaScan assay, eight were single-analyte aptasensor studies built on unmodified DNA aptamers, and one used an aptamer-conjugate imaging probe. No eligible study used an alternative multiplexed aptamer platform, and none used Spiegelmers or bead-based X-aptamers in a human aging context. This concentration is itself a finding about the state of the field rather than a selection artifact, and its consequences are discussed in Section 9.2.

### 1.3. Complementary High-Throughput Proteomic Platforms

**Figure 2:**
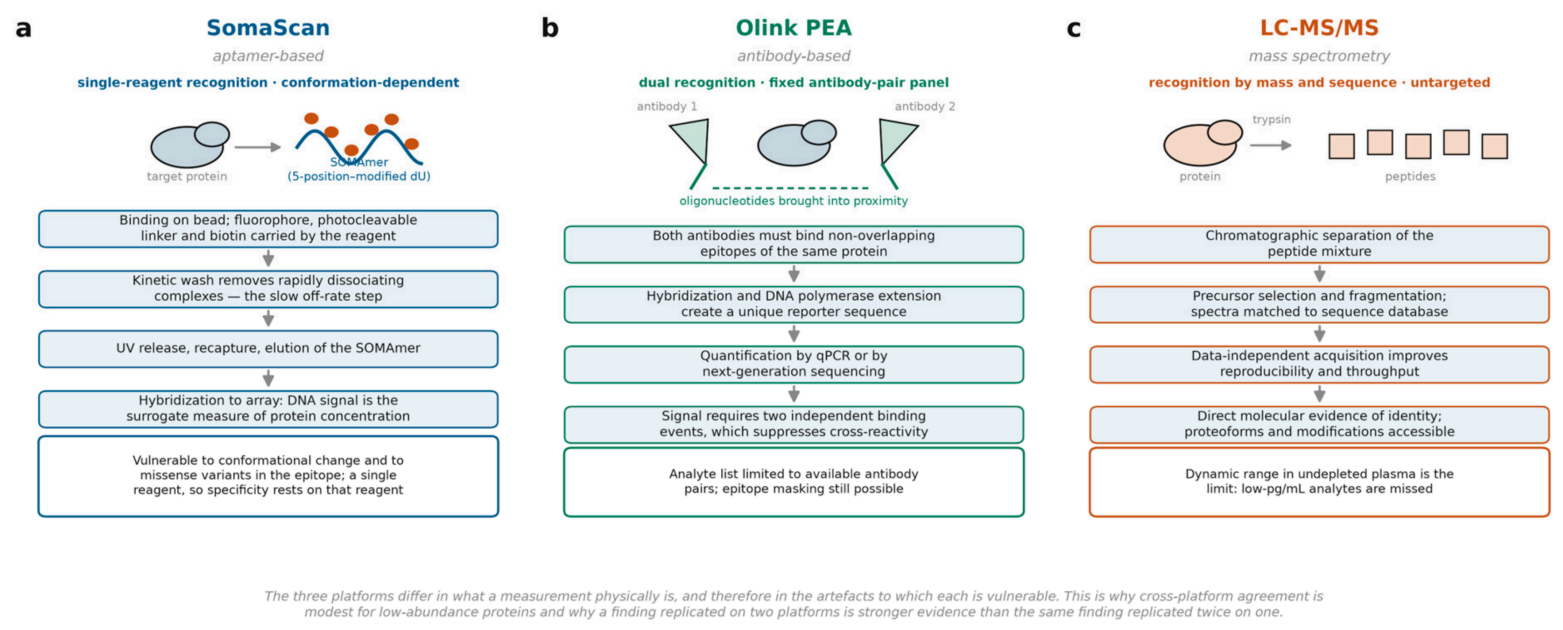
Measurement principles of the three high-throughput plasma proteomic platforms compared in this review. (**a**) SomaScan: modified aptamers (SOMAmers) bearing a fluorophore, a photocleavable linker, and a biotin tag capture target protein; a two-step catch-and-release procedure with an intervening kinetic wash removes unbound and rapidly dissociating species, and the surviving SOMAmers are quantified as a DNA surrogate on a hybridization array. Recognition is by a single reagent and depends on protein conformation. (**b**) Olink proximity extension assay: two antibodies directed at non-overlapping epitopes carry complementary oligonucleotides; only dual binding brings them into proximity, allowing extension into an amplifiable reporter read out by qPCR or sequencing. Recognition is dual and therefore more specific, but the analyte list is fixed by the available antibody pairs. (**c**) LC-MS/MS: proteins are digested to peptides, separated by chromatography and identified from their fragmentation spectra. Recognition is by mass and sequence, giving direct molecular evidence and access to proteoforms, at the cost of dynamic range in undepleted plasma.

While this review focuses on aptamer-based technologies, it is important to situate them within the broader proteomic toolkit currently used in aging research. The three platforms that dominate that toolkit—aptamer-based SomaScan, antibody-based Olink proximity extension assay (PEA), and liquid chromatography–tandem mass spectrometry (LC-MS/MS)—differ not only in coverage but in what a measurement physically is, and therefore in the artifacts to which each is vulnerable (Figure 2).

Olink PEA uses pairs of oligonucleotide-conjugated antibodies that bind to non-overlapping epitopes of the same target protein; when both bind, the attached oligonucleotides are brought into proximity, hybridize, and are extended to form a unique amplifiable reporter sequence, which is quantified by quantitative PCR or by next-generation sequencing [35,36,37]. Because signal generation requires dual recognition, the assay is highly specific and tolerates complex matrices, and the sequencing readout has allowed panel sizes to grow from 92 to several thousand analytes [37]. It has been extensively used in population-based cohort studies, including the UK Biobank and the Framingham Heart Study, to identify protein signatures of aging-related outcomes such as dementia, frailty, and cardiovascular disease [38,39]. However, PEA is restricted to a predefined panel of antibody pairs, and cross-reactivity or epitope masking can affect accuracy.

Mass spectrometry-based proteomics, particularly LC-MS/MS, provides untargeted detection of thousands of proteins, including isoforms, splice variants, and post-translational modifications, without requiring prior selection of analytes. Top-down approaches further enable detection of proteoforms—distinct protein species arising from genetic variation, alternative splicing, and post-translational modification—which are increasingly recognized as relevant to aging biology [40]. Advances in data-independent acquisition (DIA) have improved reproducibility and throughput, making MS increasingly feasible for large-scale aging studies [41,42]. MS-based studies have contributed systematic catalogs of age-associated plasma proteins and have revealed protein-level modifications in normal cognitive aging [43]. The constraint that limits MS in this particular application is dynamic range rather than resolution. The plasma proteome spans roughly ten orders of magnitude in concentration, from albumin at tens of milligrams per milliliter to regulatory cytokines below one picogram per milliliter, whereas the range accessible within a single LC-MS/MS acquisition is far narrower and is dominated by the most abundant species [12,13,14]. In practice, undepleted plasma analyzed by DIA yields a few hundred proteins drawn largely from the abundant fraction; reaching several thousand requires depletion, extensive fractionation, or nanoparticle-based enrichment, each of which costs sample volume, throughput, or both [13,42,44]. In matched specimens, neat-plasma LC-MS/MS returned on the order of 600 proteins against roughly 2600 for a high-plex affinity assay [45]. The concentrations excluded by this window are precisely those in which much of the aging signal resides: untargeted plasma MS currently operates at nanogram-per-milliliter sensitivity, whereas affinity assays operate at picogram-per-milliliter sensitivity, so cytokines such as interleukin-6—present at a few picograms per milliliter against tens of milligrams per milliliter of albumin—and cardiac troponins remain outside the routine reach of undepleted plasma MS while being standard analytes on both SomaScan and Olink panels [13,46,47]. Targeted MS assays using selected or parallel reaction monitoring with stable-isotope-labeled standards do reach these concentrations, but only for a small number of pre-specified peptides and therefore forfeit the discovery breadth that motivates untargeted MS in the first place [14]. This trade-off, rather than any general inferiority of MS, is what makes affinity platforms the pragmatic choice for hypothesis-free profiling of low-abundance circulating proteins in large aging cohorts.

Each platform has distinct strengths and limitations, and cross-platform comparisons are essential for validating findings, harmonizing results, and understanding which biological signals are robust across technologies. Direct head-to-head evaluations of aptamer-based (SomaScan), antibody-based (Olink), and MS-based platforms in neurodegenerative and aging-related cohorts remain limited in number, but a body of well-powered comparisons now exists [48,49,50,51,52,53]. These comparative analyses are discussed in Section 7.

### 1.4. Scope and Search Methodology

Forty-two original research articles met the criteria that follow. This review targets original human research, excluding reviews, meta-analyses, editorials, and conference abstracts, published from 2020 through mid-2026, in which aging-related biomarkers were measured using an aptamer-based platform (principally SOMAmer/SomaScan) or an aptamer-based biosensor. Searches were conducted in PubMed/MEDLINE and publisher databases using combinations of the terms “aptamer”, “SOMAmer” and “SomaScan” together with “aging”, “biomarker”, “senescence”, “frailty”, “longevity” and organ-specific qualifiers (cardiovascular, renal, hepatic, neurodegenerative, metabolic, musculoskeletal). Records were excluded when they relied primarily on antibody-based proximity extension assay platforms (chiefly Olink) or untargeted mass spectrometry rather than aptamers, were published before 2020, were reviews or conference abstracts without a peer-reviewed full article, or did not address human aging or aging-related disease biology. The exclusion of Olink-primary studies is a platform criterion adopted to keep the evidence base internally comparable; it is not a judgment on the quality of that literature, and its consequence—that a large part of contemporary aging proteomics falls outside this review—is examined in Section 9.2. The 42 eligible articles form the evidentiary basis of this review (Table 1). In addition to this core corpus, the review incorporates supplementary methodological and contextual works that do not strictly satisfy the selection criteria (e.g., in vitro studies, SELEX protocols, reviews on therapeutic aptamers, or analyses of pre-analytical variability) but are essential for explaining the principles of aptamer technology, interpreting platform limitations, and discussing translational perspectives.

## 2. Aptamer Technology in Biomarker Discovery

### 2.1. SELEX and Aptamer Generation for Aging-Related Targets

Original research articles describing de novo SELEX campaigns explicitly targeting proteins selected for relevance to human aging were uncommon in the post-2020 literature identified for this review. Most aptamers applied in the aging biomarker studies reviewed here were drawn from pre-existing SOMAmer libraries originally developed to cover the broad circulating proteome, and were subsequently applied in aging, frailty, and longevity cohorts. These findings suggest that the field has relied mainly on existing multiplexed aptamer libraries rather than on targeted aptamer engineering for geroscience-specific biomarkers such as Klotho, GDF-11, or senescence-specific cell-surface markers. While classical SELEX requires purified, isolated target molecules and thus necessitates bespoke purification methodologies—a particular challenge for novel or poorly characterized aging-related targets—the Cell-SELEX variant employs live cells as targets, preserving membrane proteins in their native conformation. This enables selection of aptamers against previously unknown cellular targets and can therefore support discovery of novel cell-specific biomarkers relevant to aging [94,95]. This approach has recently yielded the first reported Cell-SELEX campaign specifically targeting senescent cells: DNA aptamers selected against senescent mouse fibroblasts identified fibronectin as the molecular target, with increased aptamer staining in naturally aged mouse tissues [96]. The same strategy has been used to identify a membrane protein as a therapeutic target in cancer, illustrating that Cell-SELEX can nominate targets as well as reagents [97].

### 2.2. The SomaScan/SOMAmer Platform

The SomaScan assay (SomaLogic) is the most frequently used aptamer-based platform in the aging-related proteomic studies reviewed here [10,32,54,55,56,57,58,61,62,63,68,69,70,71,72,73,74,75,76,77,78,79,80,81,82,83]. Successive panel versions have expanded coverage from roughly 1300 to more than 7000 SOMAmer reagents, enabling simultaneous quantification of a large fraction of the detectable circulating proteome from a small volume of plasma or serum [22,27]. The platform has been used in population-based cohort studies, including the Baltimore Longitudinal Study of Aging, InCHIANTI, ARIC, the Cardiovascular Health Study, AGES-Reykjavik, and the LonGenity cohort of centenarian offspring, which generated several of the whole-plasma and organ-specific proteomic aging clocks discussed in Section 4. Methodological work has also addressed practical constraints of large-scale deployment, including the effect of long-term serum cryopreservation on SOMAmer signal stability [32] and the quantification of technical variability across panel versions [28,29].

### 2.3. Aptamer-Based Biosensors

A second, methodologically distinct body of work uses individual aptamers as recognition elements in biosensors. Here the aptamer is immobilized on a transducer rather than used in solution, and the readout is a physical signal generated when the target binds. The design constraints are consequently those of analytical chemistry rather than those of multiplexed proteomics: one analyte at a time, minutes rather than days to a result, and no requirement for a central laboratory.

Three transducer families account for the devices identified in this review. In organic electrochemical transistors, binding at a functionalized PEDOT:PSS gate modulates channel current, providing intrinsic amplification at low operating voltages [64,65]. In label-free voltammetric and impedimetric sensors, binding alters charge-transfer resistance or the current of a redox reporter at a nanostructured carbon, MXene, or gold electrode [66,67,84,85]. In optical and catalytic formats, binding is transduced through fluorescence, through mass spectrometry after aptamer-mediated capture, or through the motion of a catalytic micromotor [86,87]. Reported limits of detection lie in the picogram- to femtogram-per-milliliter range and are, in analytical terms, more than sufficient for the intended targets.

Two features of this literature should be stated at the outset because they determine how much weight the results can bear. First, the matrix in which each device was characterized varies widely, from buffer [64] through spiked serum to samples from patients [87]. Second, none of these studies followed the same individuals over time. Section 5 sets out what such devices would have to demonstrate before they could contribute to aging research; the biological significance of the individual analytes is discussed there rather than repeated here.

## 3. Aptamer-Discovered Circulating Protein Biomarkers of Human Aging

This section covers biomarkers identified by aptamer-based discovery proteomics in observational human cohorts, organized by physiological system. Two categories of study are deliberately treated elsewhere so discovery findings and application settings are not mixed: analyses performed within interventional clinical trials are described in Section 8, and single-analyte biosensor devices in Section 5. Section 3.7 sets out how the effect measures reported below should be read, and why they cannot simply be compared with one another.

### 3.1. Inflammatory and Senescence-Associated Markers

Chronic, low-grade sterile inflammation (“inflammaging”) is one of the most reproducible correlates of chronological and biological age, and elevated interleukin-6 and C-reactive protein predict mortality and disability in older adults independently of prevalent disease [98,99,100]. SomaScan-based analysis of the senescence-associated secretory phenotype (SASP) in two independent longitudinal cohorts identified GDF-15, IGFBP-2 and Cystatin-C as the SOMAmer-measured proteins most consistently associated with multiple aging traits [63]. The contribution of the multiplexed measurement here is discriminative rather than merely confirmatory: it separates SASP-associated proteins that track several aging phenotypes simultaneously from those that track only one, which no single-analyte assay can do. Aptasensors developed for individual inflammatory analytes are discussed in Section 5.

### 3.2. Metabolic and Endocrine Markers

Aptamer-based proteomics in the AGES-Reykjavik cohort of older adults identified a circulating protein signature associated with type 2 diabetes and highlighted candidate causal mediators of insulin resistance [79]. Subsequent SomaScan studies in independent cohorts identified additional predictive markers that anticipate incident type 2 diabetes years in advance and correlate with HOMA-IR-based measures of insulin resistance [80], while a further large-scale analysis combining the Cardiovascular Health Study and HERITAGE Family Study resolved plasma protein risk markers into physiologically distinct components of glucose-insulin homeostasis [81]. Collectively, these studies indicate that aptamer-based platforms can resolve metabolic aging phenotypes into mechanistically interpretable sub-signatures rather than a single undifferentiated risk score.

### 3.3. Cardiovascular Markers

The reviewed set includes several SomaScan-based studies of cardiovascular aging and cardiovascular risk in older cohorts. In the CARDIA cohort, aptamer proteomics identified circulating proteins associated with both prevalent and ten-year incident coronary artery calcification, a marker of subclinical atherosclerosis [74]. Large-scale SomaScan analyses in ARIC and HUNT identified protein networks associated with incident heart failure [75], and a related ARIC analysis found overlap between the protein pathways predicting heart failure and those predicting frailty in late life [76]. Aptamer proteomic substudies nested within the PARAGON-HF and PARADIGM-HF/ATMOSPHERE trials also identified cardiovascular risk markers, including SVEP1 [77,78]; because those analyses were performed within interventional trial programs rather than observational cohorts, they are described in Section 8.

### 3.4. Neurodegenerative and Cognitive Markers

Neurodegeneration and cognitive decline are frequent application areas in the reviewed literature. Large-scale SomaScan analyses have identified diagnostic biomarker panels and implicated pathways in Alzheimer’s disease [88], generated plasma proteomic Alzheimer’s risk scores tied to a biological age clock and a disease pseudotime trajectory [89] and, in the Whitehall II and ARIC cohorts, linked specific plasma proteins to twenty-year dementia risk [90]. A twenty-five-year follow-up study of middle-aged ARIC participants identified plasma protein markers of later-life dementia risk and implicated synaptic proteins [91], while Framingham Heart Study data yielded plasma protein risk scores for mild cognitive impairment and Alzheimer’s disease [92]. Multi-platform proteomic analysis of cerebrospinal fluid and plasma has further implicated proteostasis- and matrisome-related network biomarkers in Alzheimer’s disease [49], and an independent SomaScan study extended aptamer-based discovery proteomics to amyotrophic lateral sclerosis, identifying novel plasma proteins in affected patients [93,101]. Aptasensors targeting individual Alzheimer’s disease analytes—phosphorylated tau, total tau and amyloid-β—are a separate technology with different evidentiary requirements and are discussed in Section 5.

### 3.5. Musculoskeletal Markers: Frailty, Sarcopenia, and Bone

Physical frailty is a recurring phenotype in SomaScan proteomic studies. Work in the LonGenity cohort reported a plasma proteomic profile of frailty [68], later extended to a composite signature capturing overall health and well-being in older adults [72]. ARIC-based analyses identified late-life plasma proteins associated with prevalent and incident frailty [69] and, using earlier midlife samples from the same cohort, identified proteins measured before frailty onset that predicted late-life prefrailty and frailty [70]. A related analysis linked plasma protein signatures to objective measures of physical decline, namely gait speed and grip strength [71]. Extending this line of work to skeletal aging, a circulating proteome association study combining the Cardiovascular Health Study and the HUNT study used a SomaScan 5k panel of nearly 5000 aptamers to identify circulating proteins and pathways predicting incident hip fracture [73].

### 3.6. Renal and Hepatic Markers

Renal and hepatic aging biomarkers are less represented in the aptamer-specific literature reviewed here than cardiovascular, frailty, and neurodegenerative biomarkers. A transethnic SomaScan meta-analysis with Mendelian randomization across several European and Middle Eastern cohorts identified plasma proteins associated with estimated glomerular filtration rate, including testican-2 as a candidate causal mediator of renal function [82]. In the hepatic domain, SomaScan proteomic profiling of serum from patients with non-alcoholic steatohepatitis identified a panel of proteins differentially expressed between early- and advanced-stage liver fibrosis [83].

### 3.7. How the Reported Effect Measures Should Be Read

Associations in this literature are reported in several different metrics, and these are not interchangeable. A hazard ratio (HR) is the ratio of instantaneous event rates between the compared groups in a survival model. An HR of 2.50 for all-cause mortality [60] therefore means that, at any moment during follow-up, participants in the higher stratum of the index died at 2.5 times the rate of the reference stratum, conditional on the covariates included in that particular model. Its meaning depends entirely on three things that summary statements usually omit: the contrast on which it is computed (per standard deviation of a continuous score, per quartile, or highest versus lowest tertile), the covariate set, and the duration of follow-up. A regression coefficient behaves differently again: the value of −3.0 reported for artery AgeGap [62] is the estimated change in RBANS total score per unit of the predictor, so it is expressed in the units of one particular cognitive instrument and cannot be compared numerically with a coefficient obtained on MoCA or MMSE.

Three consequences follow for the reader. First, effect magnitudes are comparable across studies only after rescaling to a common contrast—most usefully per standard deviation of the predictor—and few of the reviewed papers report enough information to permit this. Second, when the question is clinical usefulness rather than statistical association, discrimination (C-statistic or area under the curve) and calibration are the informative quantities, and they are reported inconsistently across the corpus. Third, coefficients fitted in one cohort on one panel version rarely transfer unchanged to another, and where the reviewed studies did attempt external validation, effect sizes attenuated. For these reasons, we report effect sizes below as they were published, with their contrast where it is stated, and do not pool them. The absence of harmonized effect reporting is a substantive gap in the field rather than an editorial detail, and it is one of the specific obstacles that validation frameworks for aging biomarkers are intended to remove [15,34].

## 4. Proteomic Aging Clocks Built on Aptamer Arrays

Before discussing these models, it is important to clarify how their reported performance should be interpreted, because the reported accuracies are otherwise easy to read as self-contradictory. Proteomic clocks are supervised models trained with chronological age as the regression target. Their correlation with chronological age in a held-out sample—an *r* of 0.94, or a mean absolute error below four years—therefore measures how faithfully the model reproduced its training target. It is a descriptor of model fit, not evidence that the model measures biological aging. The quantity that carries biological information is the residual: the difference between predicted and chronological age, variously termed the age gap, AgeGap, or proteomic age acceleration. A clock is useful to geroscience precisely to the extent that this residual is associated with function, disease incidence, and mortality after adjustment for chronological age.

An important consequence of this principle is that well documented for epigenetic clocks and equally applicable here: models optimized to predict chronological age with maximum precision tend to be *worse*, not better, predictors of mortality, because the fitting procedure penalizes exactly the individual deviation that constitutes the signal of interest [102,103]. A hypothetical model that reproduced chronological age perfectly would, by construction, carry no information beyond the date of birth. Second-generation clocks were developed in response by training against composite clinical phenotypes or time to death rather than against the calendar [6,7], and the same logic governs the proteomic models reviewed below [3,104]. Correlations with chronological age are therefore quoted in this section as technical descriptors of fit; the evidence that these clocks capture aging comes from the outcome associations of their residuals, which are stated alongside [15].

### 4.1. Whole-Plasma Proteomic Age Predictors

Several studies identified in this review constructed composite proteomic age predictors from SomaScan data, in direct analogy to DNA methylation clocks. A 76-protein model derived from an approximately 1300-analyte SOMAmer panel in the InCHIANTI cohort correlated with chronological age at *r* = 0.94 in its development data [59]; the same group subsequently combined InCHIANTI and Baltimore Longitudinal Study of Aging data to show that the resulting proteomic age gap—and not the correlation itself—predicted healthspan and lifespan [10]. Analyses of the LonGenity cohort linked SomaScan-derived plasma protein profiles to age, healthspan, and all-cause mortality [54]. Comparison of centenarians with their offspring identified a protein signature consistent with slower biological aging in centenarians [55]. An unsupervised, cohort-integrated analysis of the plasma proteome across four independent cohorts identified 273 aging-associated proteins and generated a chronological-age predictor with a mean absolute error of less than four years [56]. Cross-cohort analysis of the Cardiovascular Health Study and AGES-Reykjavik also yielded a plasma proteomic signature associated with survival to advanced age [57]. A later ARIC and MESA analysis constructed midlife and late-life proteomics-based aging clocks and linked accelerated proteomic aging to elevated dementia risk [58]. Comparable proteomic clocks have been built on antibody-based platforms and validated across ancestrally diverse populations [104], which provides an external check on the biology even though those studies fall outside this review’s platform criterion.

### 4.2. Organ-Specific Proteomic Clocks

One line of work decomposes the whole-plasma proteomic aging signal into organ-specific components. Using SomaScan data from roughly 5000 proteins across approximately 6000 participants, one study derived organ-specific aging scores for eleven organs from plasma and reported that organ-specific proteomic age tracked organ-specific disease risk [61]. Subsequent work applied published organ-specific proteomic aging scores to a memory-clinic cohort and found that artery- and brain-specific proteomic age gaps were associated with cognitive performance and with risk stratification for Alzheimer’s disease and related dementias, with the artery AgeGap showing the strongest association with cognitive decline (regression coefficient −3.0 for RBANS total score; see Section 3.7 for how such coefficients should be read) [62]. These studies indicate that aptamer-based plasma proteomics can resolve some aging-associated signals at organ-enriched rather than only whole-body resolution. Complementing these cross-sectional organ clocks, a Longitudinal Proteomic Aging Index (LPAI) was developed using repeated SomaScan measurements across three visits in the ARIC study. Unlike clocks that capture cumulative aging burden at a single time point, the LPAI integrates dynamic protein trajectories through functional principal component analysis, and it is the first index in this literature to treat aging as a measured rate rather than a state. Higher LPAI was associated with increased all-cause mortality (HR = 2.50), cardiovascular mortality (HR = 1.79), and cancer mortality (HR = 1.96) [60].

### 4.3. Explainable AI and Future Directions for Proteomic Aging Models

Explainable Artificial Intelligence (XAI) methods, including SHAP (SHapley Additive exPlanations), have been applied to proteomic models to identify the proteins and pathways driving predictions, revealing biologically interpretable axes in Alzheimer’s disease such as cholinergic, tau/14–3–3, neuro-axonal, microglial/complement, and synaptic pathways [105]. These approaches consistently implicate immune regulators, extracellular-matrix proteins, and metabolic factors as key contributors, which provides a data-driven convergence with hallmarks of aging identified by other means [1]. Interpretability of this kind does not, by itself, establish causality: a protein may be a strong predictor because it lies downstream of the process of interest, and cross-sectional biomarkers of aging are in general more often consequences than causes [103].

## 5. Aptamer Biosensors for Longitudinal and Point-of-Care Monitoring

The question addressed in this section is not whether aptasensors can detect their targets—they demonstrably can—but whether the analytes they detect, and the performance they achieve, are relevant to aging research. These are separate questions, and the reviewed literature answers the first considerably better than the second.

The analytes chosen by this field are not arbitrary with respect to aging. Interleukin-6 is the best-characterized circulating correlate of inflammaging: in community-dwelling older adults, elevated IL-6 is associated with increased all-cause mortality and incident disability independently of prevalent disease, and an index combining IL-6 with soluble tumor necrosis factor receptor-1 predicts ten-year all-cause mortality [98,99,100]. C-reactive protein carries overlapping but non-identical prognostic information over comparable follow-up windows [99]. TGF-β1 participates in the fibrotic remodeling of aging tissue, and both IL-6 and TGF-β family members are components of the senescence-associated secretory phenotype through which senescent cells act on neighboring cells [63]. Total tau, phosphorylated tau, and amyloid-β constitute the blood-based neurodegeneration panel, whose concentrations are age-dependent and predictive of cognitive outcomes. The objection that IL-6 is merely an inflammation marker with no connection to aging is therefore not supported by the epidemiology. What is fair to say is that the aptasensor papers themselves seldom make this connection explicit, presenting their analyte as an analytical target rather than as a geroscience endpoint, and that this omission has obscured how directly relevant these devices could be.

Analyte relevance does not, however, make a device fit for use in aging studies. Three requirements distinguish a sensor usable in geroscience from a demonstration of analytical sensitivity, and the reviewed devices satisfy the first while largely failing to address the second and third (Table 2). (i) *Discrimination within the physiological range, not merely a low limit of detection.* Aging research needs reliable resolution of the differences that occur between older individuals, and on this criterion most of the reviewed devices fall short—in both directions. Serum interleukin-6 in community-dwelling older adults has a median near 2 pg/mL, with most values between 1 and 6 pg/mL [106]. Of the three IL-6 aptasensors reviewed, one reports a limit of detection of 16.49 pg/mL [66] and two report limits of roughly 24 pM and 100 pM [64,65], corresponding to approximately 0.6 and 2.6 ng/mL—two to three orders of magnitude above the concentrations that separate older individuals from one another. The authors of one of these studies say so themselves, noting that their device does not permit measurement under physiological conditions and is intended for the far higher concentrations reached in acute inflammation [65]. The C-reactive protein sensor has the opposite problem: its working range extends to 100 ng/mL, that is 0.1 mg/L, whereas high-sensitivity CRP in older adults is typically 1–10 mg/L [107], so samples would have to be diluted into range, and dilution was not demonstrated [67]. The aptamer-conjugated graphene oxide platform for amyloid-β1–42 operates over 0.1–10 µM [86], four to five orders of magnitude above plasma amyloid-β42 concentrations of roughly 3–11 pg/mL [108]. The devices that do match their target window are those for total tau and for amyloid-β42 oligomers [85,87], the p-tau231 sensor, whose limit of detection lies below the concentration reported in cognitively unimpaired older adults [84,109], and the TGF-β1 probe, since plasma TGF-β1 in older adults is in the low nanogram-per-milliliter range [66,110]. A low limit of detection is neither necessary nor sufficient: what matters is coverage of, and precision within, the interval in which older people actually differ, and that interval is rarely stated in these papers. (ii) *Matrix and interference.* A device characterized in buffer [64,65] has not been shown to function in a specimen containing tens of milligrams per milliliter of albumin, heterophilic antibodies, and variable lipaemia. Within the reviewed corpus, one device was evaluated in spiked human serum with acceptable recovery [84], one in plasma, cerebrospinal fluid, and brain tissue from patients with Alzheimer’s disease [87], one in three clinical sera against a single-molecule immunoassay [85], and one in senescent cells and tissue from aging mice rather than in human specimens [66]. (iii) *Stability across the measurement interval.* Longitudinal use requires that the sensor response be stable, or correctable, over days to months. Continuous electrochemical aptamer-based sensing has demonstrated precisely this capability for small molecules, with drift correction permitting uninterrupted measurement in freely moving animals [111,112], and wearable aptamer field-effect transistors have been operated on human skin [113]. Neither approach has yet been applied to a protein biomarker of aging in a human cohort.

The reviewed studies demonstrate analytical feasibility, but clinical validation remains limited. Converting these devices into instruments useful for aging research requires a different study design rather than a better limit of detection: working ranges chosen to span the physiological interval, method comparison against an established immunoassay in the same samples, within-subject reproducibility across repeated visits, and evidence that serial measurements track a functional or clinical endpoint. Of the eight aptasensor studies in the eligible set, two measured specimens from patients [85,87], one of those reported a comparison against an established immunoassay in three sera [85], and none followed the same individuals over time. This is the clearest instance in the present review of a technology whose analytical development has run well ahead of its clinical validation, and it is the reason we treat aptasensors as a promising but currently unvalidated component of the aptamer toolkit.

## 6. Senescence-Associated Biomarkers Detected via Aptamers

Cellular senescence is mechanistically linked to aging through the senescence-associated secretory phenotype (SASP), and aptamer-based methods have been applied both to quantify circulating SASP factors and to image senescent cells. At the level of circulating biomarkers, SomaScan-based analysis identified GDF-15, IGFBP-2, and Cystatin-C as SASP-associated proteins correlated with multiple aging phenotypes across two cohorts [63]. At the cellular level, an aptamer-conjugate-based ratiometric fluorescent probe was developed to image therapy-induced senescence in cancer cells, combining aptamer recognition with a senescence-associated β-galactosidase-responsive signal for dual-mode validation [114]. This imaging application was developed primarily in an oncology context; extension to non-malignant, aging-associated senescent cell populations was not identified in the peer-reviewed literature meeting this review’s criteria. The Cell-SELEX campaign against senescent fibroblasts described in Section 2.1 [96] is the most direct step toward such reagents so far, and it also indicates the route by which senescence-specific aptamers might be used for targeted delivery rather than detection alone.

## 7. Comparative Performance: Aptamer- Versus Antibody-/Mass-Spectrometry-Based Platforms

Despite the proliferation of both aptamer-based (SomaScan) and antibody-based (Olink) multiplexed proteomic platforms in aging research, head-to-head comparisons that meet this review’s platform criterion are limited in number. The principal example identified here directly compared SomaScan 7K and Olink Explore 3K measurements of cerebrospinal fluid proteins in a real-world memory-clinic cohort, reporting platform-dependent differences in analyte concordance and in the strength of association with cognitive status [48]. Comparative studies involving SomaScan 11K, Olink 5K, and mass spectrometry platforms were published in 2024–2025 [115]. To visualize the extent of cross-platform discordance, we compiled a comparative summary of studies that directly evaluated SomaScan against Olink and mass spectrometry platforms in human biospecimens (Table 3). Median correlation coefficients across platforms are consistently low to moderate (ρ ≈ 0.09–0.29), with substantial analyte-specific variability [33,48,116], a pattern reproduced in larger comparisons anchored on genetic evidence [50,51,52]. Notably, SomaScan 11K identified 279 age-associated markers absent from the 7K version and not captured by mass spectrometry, highlighting the impact of platform evolution on biomarker discovery [115].

Two questions follow directly from these data and are worth answering explicitly rather than leaving to the reader. The first is which platform has the greater predictive strength. On the available evidence, the answer is neither, and the question is better posed per analyte than per platform. Where head-to-head comparisons exist, the two affinity platforms predict clinical endpoints about equally well while disagreeing substantially about which individual proteins carry the signal [50,51,52]. Agreement is high for abundant, structurally stable analytes and poor for low-abundance ones, and the direction of superiority reverses from protein to protein: aptamer measurements are supported by *cis*-pQTL evidence for some targets where the antibody measurement is not, and the converse holds for others [50,52]. Genetic anchoring—asking whether a measurement is associated with variation at the locus encoding the protein—is currently the most objective arbiter available, and it indicates that each platform is right about a partly non-overlapping subset of the proteome. The platforms do separate cleanly in technical reproducibility rather than biology: in split plasma samples from the same participants, median coefficients of variation were 6.8% for SomaScan 11k and 35.7% for Olink Explore HT, with variability inversely related to the proportion of measurements above the limit of detection [33]. Untargeted mass spectrometry identifies fewer age-associated markers at equal sample size but supplies sequence-level evidence of what was actually measured [45,115]. The practical implication for aging research is that platform choice determines which biology is visible, and that a finding replicated on two platforms is substantially stronger evidence than the same finding replicated twice on one.

The second question is whether increased quantity has become quality. Only partly. Expanding coverage from 1.3k to 11k reagents did yield genuinely new signal, as the 279 additional age-associated markers demonstrate [115]. But the same expansion multiplies the multiple-testing burden and introduces reagents whose specificity has not been individually established, so the increase in discovered associations is not matched by an equal increase in validated ones. The reviewed corpus contains far more discovery reports than replication reports, and that ratio has not improved as panels have grown. Larger panels have therefore improved discovery breadth without yet improving evidentiary quality, and the transition from one to the other requires orthogonal validation and cross-platform replication rather than more reagents [15,117].

The limited number of such comparative studies is itself an evidence gap: cross-platform harmonization work is needed to determine how aptamer-derived and antibody-derived aging biomarker findings can be pooled, meta-analyzed, or independently replicated.

## 8. Clinical and Translational Applications

Aptamer-based proteomic platforms are being used in clinical and interventional research settings. Organ-specific proteomic aging scores have been used for dementia risk stratification in a memory-clinic cohort [62], and proteomics-based aging clocks have been evaluated for dementia risk in population cohorts [58]. Within cardiovascular medicine, aptamer proteomic substudies nested in the PARAGON-HF and PARADIGM-HF/ATMOSPHERE trial programs generated protein-based risk markers for outcomes in heart failure with preserved and with reduced ejection fraction respectively, identifying SVEP1 among others [77,78]; these analyses illustrate how aptamer platforms are deployed within clinical-trial biospecimen programs, where sample handling is standardized by protocol and outcomes are adjudicated—conditions rarely met in observational cohorts. A shared-pathway analysis linking heart-failure and frailty proteomic risk in the ARIC cohort further suggests overlap between cardiovascular and frailty-associated proteomic signals in older adults [76]. SomaScan-based proteomic profiling was also used to characterize plasma protein changes following treatment with the GLP-1 receptor agonist semaglutide in individuals with obesity, drawing on STEP 1 and STEP 2 randomized clinical trial samples [118]. This is not an aging-intervention trial, but it illustrates how aptamer-based proteomics can monitor the systemic biological effects of a pharmacological intervention relevant to metabolic and cardiovascular risk, and it is the closest existing analog to the intervention-responsiveness criterion that validation frameworks require of a biomarker of aging [15].

## 9. Challenges and Limitations

### 9.1. Cross-Reactivity, Platform Version Effects, and Validation

SOMAmer reagents, like antibodies, can exhibit off-target or conformation-dependent binding, and successive SomaScan panel versions (e.g., 1.3K, 4K, 5K, 7K, 11K) differ in reagent composition, which makes direct quantitative comparison across studies that used different panel versions non-trivial. Several of the cohort studies reviewed here explicitly reported analyses restricted to aptamer sets shared across panel versions, or flagged version-specific reagent counts, underscoring that batch and version harmonization is an active practical concern rather than a purely theoretical one [29]. Comparative studies published in 2024–2025 confirm substantial technical differences between the SomaScan and Olink platforms, as well as between different versions of each, directly affecting the reproducibility and interpretation of age-associated markers [52,115,116]. Independent biochemical or orthogonal-assay validation of individual candidate biomarkers remains inconsistent across the literature, a concern raised early in the deployment of the platform and not yet resolved [117], and relatively few studies replicate findings across ancestrally diverse cohorts.

### 9.2. Standardization, Platform Fragmentation, and Regulatory Status

A recurring issue across the reviewed literature is platform fragmentation: a substantial body of contemporary aging-proteomics research uses antibody-based Olink panels rather than aptamer-based assays, and these studies were excluded under this review’s platform criterion. This exclusion is methodologically necessary, but it illustrates the underlying problem, which is that the evidence base for proteomic biomarkers of aging is split between technologies whose measurements agree only moderately with one another [50,52]. This complicates efforts to build a unified, cross-study evidence base and motivates the comparative work discussed in Section 7. Furthermore, the aptamer-based platforms discussed in this review—both SomaScan and the standalone aptasensors—are used mainly as research tools in the reviewed studies, and none of the reviewed articles reported a regulatory-cleared aptamer assay for an aging biomarker. A further consequence of this concentration is that the field’s aptamer chemistry is narrow: essentially all multiplexed work uses one vendor’s SOMAmer library, so alternative chemistries [18,21] remain untested in aging cohorts.

### 9.3. Conformational Sensitivity and Pre-Analytical Variability

Beyond technical and market-related constraints, the interpretation of aptamer-based profiling data faces fundamental biological and pre-analytical challenges. First, SOMAmers recognize specific three-dimensional protein structures rather than merely amino acid sequences. Direct experimental evidence for this comes from studies on recombinant erythropoietin, where thermal denaturation significantly reduced aptamer binding [119]. Missense variants in surface-accessible regions of proteins can disrupt aptamer binding, and in approximately 12% of cases, platform discrepancies between SomaScan and Olink are attributable specifically to epitope accessibility [120].

Second, pre-analytical sample processing introduces systematic variability comparable to biological variation. Using neurodegenerative biomarkers as an example, all analytes exhibit substantially lower levels in serum compared with plasma, making plasma the preferred substrate [121]. Delayed processing (up to 72 h) decreases levels of Aβ40, Aβ42, NfL, and GFAP (more pronounced in plasma) but increases p-tau181 levels. Each freeze–thaw cycle reduces analyte concentrations by 9–24%. Although these data were obtained from immunoassays, they are directly relevant to aptamer platforms given similar pre-analytical requirements for target protein stability. In a broader context, levels of circulating proteins are subject to diurnal fluctuations, and circadian rhythms are considered an additional source of variability that should be accounted for when designing proteomic studies using aptamers [95]. Accounting for these factors is essential for valid cross-cohort and cross-study comparisons.

### 9.4. Cost, Access, and the Case for an Aptamer Assay Alongside Mass Spectrometry

Whether an aptamer platform is worth implementing in a setting where mass spectrometry is already available is a fair question, and the answer depends on what the laboratory is trying to do. It should first be said plainly that none of the assays reviewed here is currently a test that a patient pays for: none is regulatory-cleared for an aging indication, and all are research-use-only measurements whose costs are borne by studies rather than individuals. Per-sample costs are not systematically reported in the primary literature, but the ordering is not in dispute. At cohort scale, multiplexed affinity assays are considerably more expensive per sample than an untargeted DIA-MS acquisition on existing instrumentation, because the assay is a purchased reagent set rather than the marginal use of capital equipment [14,34,45]. Cost and reimbursement have been identified as one of the principal barriers to translating any biomarker of aging into practice, independently of the measurement technology [122]. That comparison changes once instrument capital, method development, and skilled operator time are counted, and it changes again for a laboratory that does not already operate an MS proteomics facility.

Cost, however, is not the axis on which these technologies actually compete. The case for adding an aptamer assay to an MS-equipped laboratory rests on three specific capabilities that MS does not currently deliver at cohort scale: quantification of low-abundance regulatory proteins in neat plasma without depletion or fractionation (Section 1.3); a fixed reagent set that returns the same analyte list across thousands of samples collected over many years, which is what makes longitudinal designs such as the LPAI possible at all [29,60]; and high throughput from small sample volumes, which matters for irreplaceable biobanked aliquots. Conversely, MS supplies what the aptamer platform cannot: direct sequence-level evidence that the measured species is the intended protein, access to proteoforms and post-translational modifications [40], and freedom from reagent-specific artifacts of the kind documented for epitope-disrupting missense variants [120].

The two are therefore better regarded as complementary than competing, and the strongest designs in current aging research use affinity profiling for discovery at scale and targeted MS for orthogonal confirmation of the resulting candidates. A laboratory whose question is which of several thousand proteins change with age in a given cohort is well served by an aptamer platform; one whose question is whether a specific molecule, in a specific form, is present at a specific concentration is better served by mass spectrometry. We would not recommend acquiring an aptamer assay as a second general-purpose proteomics tool unless one of the three capabilities listed above is the actual bottleneck. Until per-sample costs fall and at least one assay obtains regulatory clearance, the realistic near-term role of aptamer proteomics in aging is as a research instrument for cohort-scale discovery rather than as a clinical test offered to individual patients.

## 10. Future Perspectives

### 10.1. Computational Aptamer Design and Multi-Omic Integration

Computationally guided aptamer and SOMAmer design, together with structure-based prediction of aptamer–target binding, may help expand the repertoire of aptamers engineered specifically against aging- or senescence-defining epitopes. Several of the reviewed studies already combine aptamer-based proteomic data with clinical, cognitive, or imaging phenotypes [58,62,89]. Future studies will need to test whether integration with genomic, epigenetic, and metabolomic data in the same cohorts improves predictive accuracy or mechanistic interpretability.

### 10.2. Toward Point-of-Care and Longitudinal Aptasensing

As discussed in Section 5, existing aptasensor studies have established analytical feasibility for several individual aging-relevant analytes but have not yet produced wearable, microfluidic, or longitudinal validation in human aging cohorts. A clear translational requirement is to pair the sensing chemistries demonstrated for interleukin-6, C-reactive protein, tau, and amyloid-β [64,65,66,67,84,85,86,87] with validated sampling workflows and prospective cohort deployment.

### 10.3. Therapeutic Potential and Clinical Translation

Beyond diagnostic applications, aptamers have substantial therapeutic potential in geroscience. Their ability to bind pathological targets with high affinity—for example, aggregated proteins in neurodegenerative disease—and to act as receptor agonists or antagonists opens possibilities for targeted modulation of age-associated pathways [95]. Unlike antibodies, aptamers are chemically synthesized, show low immunogenicity, and can be chemically modified to improve pharmacokinetics, which makes them an attractive platform for geroprotective therapy [20,21,123]. Several aptamers have already reached clinical use or advanced clinical development in age-associated conditions. Pegaptanib, a PEGylated modified RNA aptamer directed against VEGF-165, was the first aptamer approved for human therapy and was licensed for neovascular age-related macular degeneration [19]. Avacincaptad pegol, an aptamer inhibitor of complement C5, was subsequently approved for geographic atrophy secondary to age-related macular degeneration on the basis of a reduction in lesion growth rate [124,125]; this is the clearest existing demonstration that aptamer chemistry can reach the clinic in an indication whose principal risk factor is age. ApTOLL, a DNA aptamer antagonist of Toll-like receptor 4, completed a first-in-human phase I study [126] and a subsequent randomized phase 1b/2a trial in acute ischaemic stroke treated by endovascular thrombectomy [127], and the program entered a development and commercialization agreement with a pharmaceutical partner in 2024. In musculoskeletal aging, aptamers directed against loop3 of sclerostin promote bone formation without the cardiovascular liability associated with antibody-mediated sclerostin blockade; the PEGylated conjugate Apc001PE received US Food and Drug Administration orphan-drug designation for osteogenesis imperfecta, and a longer-acting bimolecular conjugate subsequently received both orphan-drug and rare-pediatric-disease designations [128,129]. These programs remain preclinical, with no clinical trials reported to date, and they are cited here as evidence of regulatory feasibility rather than of clinical efficacy. In oncology, the CW06 aptamer against SLC25A24 induces senescence through disruption of methionine metabolism, establishing a metabolism–senescence axis amenable to aptamer modulation [97]. Furthermore, Cell-SELEX against senescent cells has yielded aptamers that could serve as targeting moieties for senolytic drug delivery [96]. However, as with biomarkers, translation of aptamers into clinical practice is hindered by the absence of consensus validation approaches. Systematic validation of aging biomarkers requires demonstration of prognostic validity for age-related outcomes, sensitivity to interventions, and reliability at the individual rather than merely the population level [15]. Overcoming these barriers—including the weak linkage between biomarkers and clinically meaningful decisions, high cost, and limited accessibility—remains the key challenge in moving from research discoveries to clinical application in both diagnostics and therapy.

## 11. Conclusions

Aptamer-based proteomic platforms have matured into a productive discovery engine for human aging biomarkers, delivering proteomic clocks, organ-specific risk stratification and the first dynamic measures of biological aging trajectories. The 42 original human studies (2020–2026) reviewed here collectively establish that aptamer-based platforms, particularly SomaScan, can quantify aging-associated protein signal at both systemic and organ-resolved levels, linking protein signatures to mortality, dementia, frailty and cardiovascular events.

It is worth stating concretely what this body of work has made possible that was not possible before, since the sheer number of studies can otherwise obscure the answer. Three capabilities are genuinely new. First, *organ-level resolution from a single blood draw*. Estimating how rapidly an individual’s kidney, heart or brain is aging previously required organ-specific imaging, function testing or biopsy; deriving aging scores for eleven organs from one plasma sample, and showing that each tracks disease risk in its own organ, is a capability with no prior equivalent [61], and its extension to clinical risk stratification for dementia shows that it can be acted upon [62]. Second, *measurable longitudinal trajectories*. A fixed reagent panel returning the same analyte list across repeated visits over years is what allows aging to be characterized as a rate rather than a state, and the LPAI is the first index of this kind [60]. Third, *a pre-symptomatic risk window measured in decades rather than years*. Plasma protein markers measured in midlife that predict dementia twenty to twenty-five years later [90,91], and protein signatures detectable before the onset of frailty [70], define a prevention window that no previous blood test addressed.

What has not been achieved should be stated with equal clarity. No aptamer-based aging biomarker has regulatory clearance for any aging indication. No reviewed study demonstrated that acting on a proteomic age gap changes an outcome. Discordance between platforms remains unresolved, pre-analytical variability is under-reported, replication across ancestrally diverse cohorts is sparse, and the aptasensor literature has not yet produced a single longitudinal human measurement. The field has generated a large and internally coherent body of discovery and comparatively little validation, and closing that gap, rather than further expanding panel size, is what will determine whether these measurements become clinically useful.

Several critical gaps must therefore be addressed before these biomarkers can enter routine clinical practice. Cross-platform discordance between SomaScan, Olink and mass spectrometry hampers data harmonization and meta-analysis. Pre-analytical variability, including sample type, freeze–thaw cycles and circadian fluctuation, is often under-reported, yet it can be comparable in magnitude to the biological signal. Cost and the research-use-only status of the assays further restrict access outside well-funded cohort studies.

Looking forward, we identify four priority actions:

(1) systematic head-to-head comparisons and the development of cross-platform calibration standards;
(2) targeted SELEX campaigns against senescence-specific epitopes (e.g., SASP factors) to move beyond generic proteome coverage;
(3) prospective validation of aptasensors in real-world aging cohorts, coupled with wearable or microfluidic integration for remote monitoring; and
(4) XAI-driven interpretability, multi-omics integration, and deeper resolution at tissue- and cell-type-specific levels.

With concerted effort on validation, standardization, and translation—and with the reporting discipline described in Section 3.7—aptamer-based biomarkers are well positioned to move from research tools to clinical instruments for risk stratification, intervention monitoring, and personalized geroprotective medicine.

## Figures and Tables

**Table 1:** Overview of aptamer/SOMAmer-detected biomarker categories in human aging.

Biomarker Category	Representative Markers	Platform	Representative Studies
Whole-plasma proteomic age	Multi-protein composite scores	SomaScan 1.3k–7k	[10,54,55,56,57,58]
High-correlation proteomic age	76-protein panel	SomaScan 1.3k	[59]
Longitudinal Proteomic Aging Index (LPAI)	Dynamic protein trajectories (functional PCA)	SomaScan 5k	[60]
Organ-specific aging	Organ-weighted protein panels (heart, kidney, brain, etc.)	SomaScan 4k–5k; organ-weighted models	[61,62]
Inflammaging/SASP	GDF-15, IL-6, IGFBP-2, Cystatin-C, CRP	SomaScan; electrochemical aptasensors	[63,64,65,66,67]
Frailty/sarcopenia/bone	Gait speed/grip strength panels, hip-fracture risk proteins	SomaScan 4k–5k	[68,69,70,71,72,73]
Cardiovascular aging	SVEP1, coronary calcium panel, heart-failure risk proteins	SomaScan 4k–7k	[74,75,76,77,78]
Metabolic/endocrine aging	ACY1, WFIKKN2, T2D risk proteins	SomaScan 4k	[79,80,81]
Renal/hepatic aging	Testican-2, eGFR-associated proteins, NASH fibrosis panel	SomaScan	[82,83]
Neurodegeneration/cognition	p-tau231, Aβ1–42, tau, synaptic proteins	SomaScan; electrochemical/optical aptasensors	[49,84,85,86,87,88,89,90,91,92,93]

**Table 2:** Aptamer-based biosensors for aging-relevant analytes identified in this review: analytical performance as reported by the authors, and the matrix in which each device was characterized. Sensitivity is uniformly adequate; validation in human specimens, and in particular repeated measurement in the same individuals, is not.

Analyte	Sensing Format	Limit of Detection (as Reported)	Working Range (as Reported)	Matrix in Which Characterized	Concentration in Older Adults	Ref.
TGF-β1	Cu-loaded semiconducting polymer dots; wireless resistive readout	193.09 pg/mL	Not reported	Senescent cells; serum and tissue from aging mice (6–20 months); no human specimens	Plasma ≈ 2–6 ng/mL [110]	[66]
IL-6	Cu-loaded semiconducting polymer dots; wireless resistive readout	16.49 pg/mL	Not reported	Senescent cells; serum and tissue from aging mice (6–20 months); no human specimens	Serum median ≈ 2.3 pg/mL [106]	[66]
IL-6	Organic electrochemical transistor; AuNP-functionalized PEDOT:PSS gate	≈24 pM (≈0.6 ng/mL)	10 pM–500 nM	Phosphate-buffered saline only; selectivity tested against TNF	Serum median ≈ 2.3 pg/mL [106]	[64]
IL-6	Organic electrochemical transistor; functionalized PEDOT:PSS	100 pM (≈2.6 ng/mL)	10–1000 pM	Buffer; spiked bovine plasma (threshold rose to ≈1 nM)	Serum median ≈ 2.3 pg/mL [106]	[65]
C-reactive protein	Label-free differential pulse voltammetry; AuNPs/carboxylated graphene oxide	2.9 × 10^−4^ ng/mL	0.001–100 ng/mL	Buffer; artificial serum spikes	High-sensitivity CRP ≈ 1–10 mg/L [107]	[67]
p-tau231	Electrochemical; AuNPs on glassy carbon electrode	2.31 pg/mL	10–10^7^ pg/mL	Spiked human serum (recovery 97.6–103.3%)	≈15 pg/mL if cognitively unimpaired; ≈29 pg/mL in Alzheimer’s disease [109]	[84]
Amyloid-β1–42	Aptamer-conjugated graphene oxide with SALDI mass spectrometry	≈0.1 µM (≈450 ng/mL)	0.1–10 µM	Spiked human serum; SH-SY5Y cell model	Plasma ≈ 3–11 pg/mL [108]	[86]
Amyloid-β42 oligomers	Catalytic micromotor; graphene oxide–AuNPs, label-free electrochemical	0.10 pg/mL	0.5–500 pg/mL	Brain tissue, cerebrospinal fluid and plasma from patients with Alzheimer’s disease (5 µL, undiluted)	No established reference interval for oligomers	[87]
Total tau	Portable vertical graphene@Au electrochemical platform	0.034 pg/mL	0.1 pg/mL–1 ng/mL	Buffer calibration; three clinical sera, compared with a single-molecule immunoassay	Plasma ≈ 0.2–3.1 pg/mL [108]	[85]

Two of the nine device–analyte combinations were evaluated in specimens from patients, and only one reported a comparison against an established immunoassay; none was applied to repeated samples from the same individuals. Molar limits of detection have been converted to mass concentration using the molecular weight of the mature protein. Values are quoted as published and are not directly comparable across formats.

**Table 3:** Comparative performance of aptamer-based (SomaScan), antibody-based (Olink), and mass spectrometry platforms in human proteomic studies of aging.

Platform Comparison	Sample Type	Number of Analytes (Overlap)	Key Observations	Reference
SomaScan 7K vs. Olink Explore 3K	CSF (memory clinic cohort)	~2500	More than 600 proteins were reproducibly measured by Olink; the strength of association with cognitive status varied significantly between platforms.	[48]
SomaScan 11K vs. Olink 5K	Plasma	~4500	The distribution of between-platform correlations was bimodal, with peaks near r = 0 and r = 0.8.	[33]
SomaScan 7K/11K vs. Olink 3K/5K vs. MS	Plasma	Varies by platform	SomaScan 11K and Olink 5K identified a broader range of age-associated markers than their predecessor platforms; MS platforms identified fewer significant markers.	[115]
SomaScan 7K vs. Olink Explore 3K	Plasma (4000 adults)	2168 (overlapping assays)	Median between-platform correlation was modest (ρ = 0.29). Proteins associated with body-mass index and with ischaemic heart disease risk were identified, and adding protein measurements to conventional risk factors improved prediction.	[116]

## Data Availability

No new data were created or analyzed in this study. Data sharing is not applicable to this article.

## References

[1] López-Otín C., Blasco M.A., Partridge L., Serrano M., Kroemer G. (2023). Hallmarks of aging: An expanding universe. Cell.

[2] Ferrucci L., Gonzalez-Freire M., Fabbri E., Simonsick E., Tanaka T., Moore Z., Salimi S., Sierra F., de Cabo R. (2020). Measuring biological aging in humans: A quest. Aging Cell.

[3] Rutledge J., Oh H., Wyss-Coray T. (2022). Measuring biological age using omics data. Nat. Rev. Genet..

[4] Horvath S. (2013). DNA methylation age of human tissues and cell types. Genome Biol..

[5] Hannum G., Guinney J., Zhao L., Zhang L., Hughes G., Sadda S., Klotzle B., Bibikova M., Fan J.B., Gao Y. (2013). Genome-wide Methylation Profiles Reveal Quantitative Views of Human Aging Rates. Mol. Cell.

[6] Levine M.E., Lu A.T., Quach A., Chen B.H., Assimes T.L., Bandinelli S., Hou L., Baccarelli A.A., Stewart J.D., Li Y. (2018). An epigenetic biomarker of aging for lifespan and healthspan. Aging.

[7] Lu A.T., Quach A., Wilson J.G., Reiner A.P., Aviv A., Raj K., Hou L., Baccarelli A.A., Li Y., Stewart J.D. (2019). DNA methylation GrimAge strongly predicts lifespan and healthspan. Aging.

[8] Peters M.J., Joehanes R., Pilling L.C., Schurmann C., Conneely K.N., Powell J., Reinmaa E., Sutphin G.L., Zhernakova A., Schramm K. (2015). The transcriptional landscape of age in human peripheral blood. Nat. Commun..

[9] Menni C., Kastenmüller G., Petersen A.K., Bell J.T., Psatha M., Tsai P.C., Gieger C., Schulz H., Erte I., John S. (2013). Metabolomic markers reveal novel pathways of ageing and early development in human populations. Int. J. Epidemiol..

[10] Tanaka T., Basisty N., Fantoni G., Candia J., Moore A.Z., Biancotto A., Schilling B., Bandinelli S., Ferrucci L. (2020). Plasma proteomic biomarker signature of age predicts health and life span. eLife.

[11] Williams S.A., Kivimaki M., Langenberg C., Hingorani A.D., Casas J.P., Bouchard C., Jonasson C., Sarzynski M.A., Shipley M.J., Alexander L. (2019). Plasma protein patterns as comprehensive indicators of health. Nat. Med..

[12] Anderson N.L., Anderson N.G. (2002). The Human Plasma Proteome: History, Character, and Diagnostic Prospects. Mol. Cell. Proteom..

[13] Geyer P.E., Holdt L.M., Teupser D., Mann M. (2017). Revisiting biomarker discovery by plasma proteomics. Mol. Syst. Biol..

[14] Deutsch E.W., Omenn G.S., Sun Z., Maes M., Pernemalm M., Palaniappan K.K., Letunica N., Vandenbrouck Y., Brun V., Tao S.C. (2021). Advances and Utility of the Human Plasma Proteome. J. Proteome Res..

[15] Moqri M., Herzog C., Poganik J.R., Ying K., Justice J.N., Belsky D.W., Higgins-Chen A.T., Chen B.H., Cohen A.A., Fuellen G. (2024). Validation of biomarkers of aging. Nat. Med..

[16] Tuerk C., Gold L. (1990). Systematic Evolution of Ligands by Exponential Enrichment: RNA Ligands to Bacteriophage T4 DNA Polymerase. Science.

[17] Ellington A.D., Szostak J.W. (1990). In vitro selection of RNA molecules that bind specific ligands. Nature.

[18] Dunn M.R., Jimenez R.M., Chaput J.C. (2017). Analysis of aptamer discovery and technology. Nat. Rev. Chem..

[19] Gragoudas E.S., Adamis A.P., Cunningham E.T., Feinsod M., Guyer D.R. (2004). Pegaptanib for Neovascular Age-Related Macular Degeneration. N. Engl. J. Med..

[20] Keefe A.D., Pai S., Ellington A. (2010). Aptamers as therapeutics. Nat. Rev. Drug Discov..

[21] Zhou J., Rossi J. (2017). Aptamers as targeted therapeutics: Current potential and challenges. Nat. Rev. Drug Discov..

[22] Gold L., Ayers D., Bertino J., Bock C., Bock A., Brody E.N., Carter J., Dalby A.B., Eaton B.E., Fitzwater T. (2010). Aptamer-Based Multiplexed Proteomic Technology for Biomarker Discovery. PLoS ONE.

[23] Vaught J.D., Bock C., Carter J., Fitzwater T., Otis M., Schneider D., Rolando J., Waugh S., Wilcox S.K., Eaton B.E. (2010). Expanding the Chemistry of DNA for in Vitro Selection. J. Am. Chem. Soc..

[24] Rohloff J.C., Gelinas A.D., Jarvis T.C., Ochsner U.A., Schneider D.J., Gold L., Janjic N. (2014). Nucleic Acid Ligands With Protein-like Side Chains: Modified Aptamers and Their Use as Diagnostic and Therapeutic Agents. Mol. Ther. Nucleic Acids.

[25] Davies D.R., Gelinas A.D., Zhang C., Rohloff J.C., Carter J.D., O’Connell D., Waugh S.M., Wolk S.K., Mayfield W.S., Burgin A.B. (2012). Unique motifs and hydrophobic interactions shape the binding of modified DNA ligands to protein targets. Proc. Natl. Acad. Sci. USA.

[26] Gelinas A.D., Davies D.R., Janjic N. (2016). Embracing proteins: Structural themes in aptamer-protein complexes. Curr. Opin. Struct. Biol..

[27] Kraemer S., Schneider D.J., Paterson C., Perry D., Westacott M.J., Hagar Y., Katilius E., Lynch S., Russell T.M., Johnson T. (2024). Crossing the Halfway Point: Aptamer-Based, Highly Multiplexed Assay for the Assessment of the Proteome. J. Proteome Res..

[28] Candia J., Cheung F., Kotliarov Y., Fantoni G., Sellers B., Griesman T., Huang J., Stuccio S., Zingone A., Ryan B.M. (2017). Assessment of Variability in the SOMAscan Assay. Sci. Rep..

[29] Candia J., Daya G.N., Tanaka T., Ferrucci L., Walker K.A. (2022). Assessment of variability in the plasma 7k SomaScan proteomics assay. Sci. Rep..

[30] Emilsson V., Ilkov M., Lamb J.R., Finkel N., Gudmundsson E.F., Pitts R., Hoover H., Gudmundsdottir V., Horman S.R., Aspelund T. (2018). Co-regulatory networks of human serum proteins link genetics to disease. Science.

[31] Sun B.B., Maranville J.C., Peters J.E., Stacey D., Staley J.R., Blackshaw J., Burgess S., Jiang T., Paige E., Surendran P. (2018). Genomic atlas of the human plasma proteome. Nature.

[32] Beppo R., Ohashi Y., Yamamoto K., Kinoshita F., Kato T.S., Katsuno M., Matsubara T., Yokota M., Ichihara S., Nakatochi M. (2025). Evaluation of serum samples in long cryopreservation for SomaScan proteomics and sex differences in elderly Japanese adults. Sci. Rep..

[33] Rooney M.R., Chen J., Ballantyne C.M., Hoogeveen R.C., Boerwinkle E., Yu B., Walker K.A., Schlosser P., Selvin E., Chatterjee N. (2025). Correlations Within and Between Highly Multiplexed Proteomic Assays of Human Plasma. Clin. Chem..

[34] Suhre K., McCarthy M.I., Schwenk J.M. (2021). Genetics meets proteomics: Perspectives for large population-based studies. Nat. Rev. Genet..

[35] Lundberg M., Eriksson A., Tran B., Assarsson E., Fredriksson S. (2011). Homogeneous antibody-based proximity extension assays provide sensitive and specific detection of low-abundant proteins in human blood. Nucleic Acids Res..

[36] Assarsson E., Lundberg M., Holmquist G., Björkesten J., Bucht Thorsen S., Ekman D., Eriksson A., Rennel Dickens E., Ohlsson S., Edfeldt G. (2014). Homogenous 96-Plex PEA Immunoassay Exhibiting High Sensitivity, Specificity, and Excellent Scalability. PLoS ONE.

[37] Wik L., Nordberg N., Broberg J., Björkesten J., Assarsson E., Henriksson S., Grundberg I., Pettersson E., Westerberg C., Liljeroth E. (2021). Proximity Extension Assay in Combination with Next-Generation Sequencing for High-throughput Proteome-wide Analysis. Mol. Cell. Proteom..

[38] Gadd D.A., Hillary R.F., Kuncheva Z., Mangelis T., Cheng Y., Dissanayake M., Admanit R., Gagnon J., Lin T., Ferber K.L. (2024). Blood protein assessment of leading incident diseases and mortality in the UK Biobank. Nat. Aging.

[39] Kuo C.L., Liu P., Drouard G., Vuoksimaa E., Kaprio J., Ollikainen M., Chen Z., Pilling L.C., Atkins J.L., Fortinsky R.H. (2025). A proteomic signature of healthspan. Proc. Natl. Acad. Sci. USA.

[40] Toby T.K., Fornelli L., Kelleher N.L. (2016). Progress in Top-Down Proteomics and the Analysis of Proteoforms. Annu. Rev. Anal. Chem..

[41] Kliuchnikova A.A., Ilgisonis E.V., Archakov A.I., Ponomarenko E.A., Moskalev A.A. (2024). Proteomic Markers of Aging and Longevity: A Systematic Review. Int. J. Mol. Sci..

[42] Messner C.B., Demichev V., Bloomfield N., Yu J.S.L., White M., Kreidl M., Egger A.S., Freiwald A., Ivosev G., Wasim F. (2021). Ultra-fast proteomics with Scanning SWATH. Nat. Biotechnol..

[43] Kamalian A., Shichkova P., Tognetti M., Below C., Ho S.G., Bruderer R., Reiter L., Feng Y., Lutz M.W., Moghekar A. (2025). Unveiling proteomic and peptide-level modifications in cerebrospinal fluid and plasma in normal cognitive aging. Commun. Med..

[44] Blume J.E., Manning W.C., Troiano G., Hornburg D., Figa M., Hesterberg L., Platt T.L., Zhao X., Cuaresma R.A., Everley P.A. (2020). Rapid, deep and precise profiling of the plasma proteome with multi-nanoparticle protein corona. Nat. Commun..

[45] Beimers W.F., Overmyer K.A., Sinitcyn P., Lancaster N.M., Quarmby S.T., Coon J.J. (2025). Technical Evaluation of Plasma Proteomics Technologies. J. Proteome Res..

[46] Zhu Y. (2024). Plasma/Serum Proteomics based on Mass Spectrometry. Protein Pept. Lett..

[47] Smith J.G., Gerszten R.E. (2017). Emerging Affinity-Based Proteomic Technologies for Large-Scale Plasma Profiling in Cardiovascular Disease. Circulation.

[48] Puerta R., Cano A., García-González P., García-Gutiérrez F., Capdevila M., de Rojas I., Olivé C., Blázquez-Folch J., Sotolongo-Grau O., Miguel A. (2025). Head-to-Head Comparison of Aptamer- and Antibody-Based Proteomic Platforms in Human Cerebrospinal Fluid Samples from a Real-World Memory Clinic Cohort. Int. J. Mol. Sci..

[49] Dammer E.B., Ping L., Duong D.M., Modeste E.S., Seyfried N.T., Lah J.J., Levey A.I., Johnson E.C.B. (2022). Multi-platform proteomic analysis of Alzheimer’s disease cerebrospinal fluid and plasma reveals network biomarkers associated with proteostasis and the matrisome. Alzheimer’s Res. Ther..

[50] Pietzner M., Wheeler E., Carrasco-Zanini J., Kerrison N.D., Oerton E., Koprulu M., Luan J., Hingorani A.D., Williams S.A., Wareham N.J. (2021). Synergistic insights into human health from aptamer- and antibody-based proteomic profiling. Nat. Commun..

[51] Katz D.H., Robbins J.M., Deng S., Tahir U.A., Bick A.G., Pampana A., Yu Z., Ngo D., Benson M.D., Chen Z.Z. (2022). Proteomic profiling platforms head to head: Leveraging genetics and clinical traits to compare aptamer- and antibody-based methods. Sci. Adv..

[52] Eldjarn G.H., Ferkingstad E., Lund S.H., Helgason H., Magnusson O.T., Gunnarsdottir K., Olafsdottir T.A., Halldorsson B.V., Olason P.I., Zink F. (2023). Large-scale plasma proteomics comparisons through genetics and disease associations. Nature.

[53] Raffield L.M., Dang H., Pratte K.A., Jacobson S., Gillenwater L.A., Ampleford E., Barjaktarevic I., Basta P., Clish C.B., Comellas A.P. (2020). Comparison of Proteomic Assessment Methods in Multiple Cohort Studies. Proteomics.

[54] Sathyan S., Ayers E., Gao T., Weiss E.F., Milman S., Verghese J., Barzilai N. (2020). Plasma proteomic profile of age, health span, and all-cause mortality in older adults. Aging Cell.

[55] Sebastiani P., Federico A., Morris M., Gurinovich A., Tanaka T., Chandler K.B., Andersen S.L., Denis G., Costello C.E., Ferrucci L. (2021). Protein signatures of centenarians and their offspring suggest centenarians age slower than other humans. Aging Cell.

[56] Coenen L., Lehallier B., de Vries H.E., Middeldorp J. (2023). Markers of aging: Unsupervised integrated analyses of the human plasma proteome. Front. Aging.

[57] Liu X., Axelsson G.T., Newman A.B., Psaty B.M., Boudreau R.M., Wu C., Arnold A.M., Aspelund T., Austin T.R., Gardin J.M. (2024). Plasma proteomic signature of human longevity. Aging Cell.

[58] Sedaghat S., Park S., Walker R.F., Wang S., Liu J., Hughes T.M., Sabayan B., Tang W., Coresh J., Pankow J.S. (2025). Proteomics-based aging clocks in midlife or late-life and their associated risk of dementia. Commun. Med..

[59] Tanaka T., Biancotto A., Moaddel R., Moore A.Z., Gonzalez-Freire M., Aon M.A., Candia J., Zhang P., Cheung F., Fantoni G. (2018). Plasma proteomic signature of age in healthy humans. Aging Cell.

[60] Rao Z., Wang S., Li A., Blaha M.J., Coresh J., Ganz P., Marshall C.H., Pankow J.S., Platz E.A., Post W. (2026). A Novel Longitudinal Proteomic Aging Index Predicts Mortality, Multimorbidity, and Frailty in Older Adults. Aging Cell.

[61] Oh H.S.H., Rutledge J., Nachun D., Pálovics R., Abiose O., Moran-Losada P., Channappa D., Urey D.Y., Kim K., Sung Y.J. (2023). Organ aging signatures in the plasma proteome track health and disease. Nature.

[62] Kang S., Baker S., Hayhoe B., Price G., Novak G., Wong J., Middleton L., Robinson O. (2025). Organ-specific proteomic aging and cognitive performance: Implications for risk prediction of Alzheimer’s disease and related dementias in older adults. J. Prev. Alzheimer’s Dis..

[63] Evans D.S., Young D., Tanaka T., Basisty N., Bandinelli S., Ferrucci L., Campisi J., Schilling B. (2024). Proteomic Analysis of the Senescence-Associated Secretory Phenotype: GDF-15, IGFBP-2, and Cystatin-C Are Associated With Multiple Aging Traits. J. Gerontol. A Biol. Sci. Med. Sci..

[64] Diacci C., Burtscher B., Berto M., Ruoko T.P., Lienemann S., Greco P., Berggren M., Borsari M., Simon D.T., Bortolotti C.A. (2024). Organic Electrochemical Transistor Aptasensor for Interleukin-6 Detection. ACS Appl. Mater. Interfaces.

[65] Burtscher B., Diacci C., Makhinia A., Savvakis M., Gabrielsson E.O., Veith L., Liu X., Strakosas X., Simon D.T. (2024). Functionalization of PEDOT:PSS for aptamer-based sensing of IL6 using organic electrochemical transistors. npj Biosens..

[66] Robby A.I., Jiang S., Jin E.J., Park S.Y. (2025). Semiconducting polymer dot-based wireless electrochemical aptasensor for detection of aging-related TGF-β1 and IL-6. Anal. Chim. Acta.

[67] Gao H., Bai Y., He B., Tan C.S. (2022). A Simple Label-Free Aptamer-Based Electrochemical Biosensor for the Sensitive Detection of C-Reactive Proteins. Biosensors.

[68] Sathyan S., Ayers E., Gao T., Milman S., Barzilai N., Verghese J. (2020). Plasma proteomic profile of frailty. Aging Cell.

[69] Liu F., Austin T.R., Schrack J.A., Chen J., Walston J., Mathias R.A., Grams M., Odden M.C., Newman A., Psaty B.M. (2023). Late-life plasma proteins associated with prevalent and incident frailty: A proteomic analysis. Aging Cell.

[70] Liu F., Schrack J.A., Walston J., Mathias R.A., Windham B.G., Grams M.E., Coresh J., Walker K.A. (2024). Mid-life plasma proteins associated with late-life prefrailty and frailty: A proteomic analysis. GeroScience.

[71] Liu X., Pan S., Xanthakis V., Vasan R.S., Psaty B.M., Austin T.R., Newman A.B., Sanders J.L., Wu C., Tracy R.P. (2022). Plasma proteomic signature of decline in gait speed and grip strength. Aging Cell.

[72] Sathyan S., Liu F., Tanaka T., Ferrucci L., Ayers E., Windham B.G., Gao T., Weiss E.F., Candia J., Coresh J. (2025). A Frailty-Based Plasma Proteomic Signature Capturing Overall Health and Well-Being in Older Adults. Aging Cell.

[73] Austin T.R., Fink H.A., Jalal D.I., Törnqvist A.E., Buzkova P., Barzilay J.I., Lu T., Carbone L., Gabrielsen M.E., Grahnemo L. (2024). Large-scale circulating proteome association study (CPAS) meta-analysis identifies circulating proteins and pathways predicting incident hip fractures. J. Bone Miner. Res..

[74] El-Sabawi B., Huang X., Lin P., Anwar M.Y., Betti M., Kim N., Perry A.S., Perera B.L.A., Gajjar P., Colangelo L.A. (2026). Proteogenomic Analysis of Coronary Artery Calcification in Human Populations. Arterioscler. Thromb. Vasc. Biol..

[75] Shah A.M., Myhre P.L., Arthur V., Dorbala P., Rasheed H., Buckley L.F., Claggett B., Liu G., Ma J., Nguyen N.Q. (2024). Large scale plasma proteomics identifies novel proteins and protein networks associated with heart failure development. Nat. Commun..

[76] Ramonfaur D., Buckley L.F., Arthur V., Yang Y., Claggett B.L., Ndumele C.E., Walker K.A., Austin T., Odden M.C., Floyd J.S. (2024). High Throughput Plasma Proteomics and Risk of Heart Failure and Frailty in Late Life. JAMA Cardiol..

[77] Patel-Murray N.L., Zhang L., Claggett B.L., Xu D., Serrano-Fernandez P., Healey M., Wandel S., Chen C., Jacob J., Xu H. (2024). Aptamer Proteomics for Biomarker Discovery in Heart Failure With Preserved Ejection Fraction: The PARAGON-HF Proteomic Substudy. J. Am. Heart Assoc..

[78] Zhang L., Cunningham J.W., Claggett B.L., Jacob J., Mendelson M.M., Serrano-Fernandez P., Kaiser S., Yates D.P., Healey M., Chen C.W. (2022). Aptamer Proteomics for Biomarker Discovery in Heart Failure With Reduced Ejection Fraction. Circulation.

[79] Gudmundsdottir V., Zaghlool S.B., Emilsson V., Aspelund T., Ilkov M., Gudmundsson E.F., Jonsson S.M., Zilhão N.R., Lamb J.R., Suhre K. (2020). Circulating Protein Signatures and Causal Candidates for Type 2 Diabetes. Diabetes.

[80] Ngo D., Benson M.D., Long J.Z., Chen Z.Z., Wang R., Nath A.K., Keyes M.J., Shen D., Sinha S., Kuhn E. (2021). Proteomic profiling reveals biomarkers and pathways in type 2 diabetes risk. JCI Insight.

[81] Cronjé H.T., Mi M.Y., Austin T.R., Biggs M.L., Siscovick D.S., Lemaitre R.N., Psaty B.M., Tracy R.P., Djoussé L., Kizer J.R. (2023). Plasma Proteomic Risk Markers of Incident Type 2 Diabetes Reflect Physiologically Distinct Components of Glucose-Insulin Homeostasis. Diabetes.

[82] Matías-García P.R., Wilson R., Guo Q., Zaghlool S.B., Eales J.M., Xu X., Charchar F.J., Dormer J., Maalmi H., Schlosser P. (2021). Plasma Proteomics of Renal Function: A Transethnic Meta-Analysis and Mendelian Randomization Study. J. Am. Soc. Nephrol..

[83] Luo Y., Wadhawan S., Greenfield A., Decato B.E., Oseini A.M., Collen R., Shevell D.E., Thompson J., Jarai G., Charles E.D. (2021). SOMAscan Proteomics Identifies Serum Biomarkers Associated with Liver Fibrosis in Patients With NASH. Hepatol. Commun..

[84] Kong Q., Liu C., Zhang Y., He Y., Zhang R., Wang Y., Zhou Q., Cui F. (2024). Nucleic acid aptamer-based electrochemical sensor for the detection of serum P-tau231 and the instant screening test of Alzheimer’s disease. Microchim. Acta.

[85] Liu Y., Liu X., Li M., Liu Q., Xu T. (2022). Portable Vertical Graphene@Au-Based Electrochemical Aptasensing Platform for Point-of-Care Testing of Tau Protein in the Blood. Biosensors.

[86] Song G., Shui R., Wang D., Fang R., Yuan T., Li L., Feng J., Gao F., Shen Q., Gong J. (2022). Aptamer-conjugated graphene oxide-based surface assisted laser desorption ionization mass spectrometry for selective extraction and detection of Aβ1--42 in an Alzheimer’s disease SH-SY5 cell model. Front. Aging Neurosci..

[87] Gallo-Orive Á., Moreno-Guzmán M., Sanchez-Paniagua M., Montero-Calle A., Barderas R., Escarpa A. (2024). Gold Nanoparticle-Decorated Catalytic Micromotor-Based Aptassay for Rapid Electrochemical Label-Free Amyloid-β42 Oligomer Determination in Clinical Samples from Alzheimer’s Patients. Anal. Chem..

[88] Heo G., Xu Y., Wang E., Ali M., Oh H.S.H., Moran-Losada P., Anastasi F., González Escalante A., Puerta R., Song S. (2025). Large-scale plasma proteomic profiling unveils diagnostic biomarkers and pathways for Alzheimer’s disease. Nat. Aging.

[89] Liu S., Huang Y., Park T., Lee E.H., Chaudhuri S., Adzibolosu N., Bice P.J., Dage J.L., Brosch J.R., Gao S. (2025). Plasma proteomic Alzheimer’s risk score: Biological age clock and pseudotime trajectory. Alzheimer’s Dement..

[90] Lindbohm J.V., Mars N., Walker K.A., Singh-Manoux A., Livingston G., Brunner E.J., Sipilä P.N., Saksela K., Ferrie J.E., Lovering R.C. (2022). Plasma proteins, cognitive decline, and 20-year risk of dementia in the Whitehall II and Atherosclerosis Risk in Communities studies. Alzheimer’s Dement..

[91] Walker K.A., Chen J., Shi L., Yang Y., Fornage M., Zhou L., Schlosser P., Surapaneni A., Grams M.E., Duggan M.R. (2023). Proteomics analysis of plasma from middle-aged adults identifies protein markers of dementia risk in later life. Sci. Transl. Med..

[92] Rehman H., Ang T.F.A., Tao Q., Au R., Farrer L.A., Qiu W.Q., Zhang X. (2025). Plasma protein risk scores for mild cognitive impairment and Alzheimer’s disease in the Framingham heart study. Alzheimer’s Dement..

[93] Berrone E., Chiorino G., Guana F., Benedetti V., Palmitessa C., Gallo M., Calvo A., Casale F., Manera U., Favole A. (2023). SOMAscan Proteomics Identifies Novel Plasma Proteins in Amyotrophic Lateral Sclerosis Patients. Int. J. Mol. Sci..

[94] Sefah K., Shangguan D., Xiong X., O’Donoghue M.B., Tan W. (2010). Development of DNA aptamers using Cell-SELEX. Nat. Protoc..

[95] Park T.-I., Yang A.H., Kanth B.K., Pack S.P. (2025). Aptamers as Diagnostic and Therapeutic Agents for Aging and Age-Related Diseases. Biosensors.

[96] Pearson K.S., Jachim S.K., Doherty C.D., Wilbanks B.A., Prieto L.I., Dugan M., Baker D.J., LeBrasseur N.K., Maher L.J. (2025). An Unbiased Cell-Culture Selection Yields DNA Aptamers as Novel Senescent Cell-Specific Reagents. Aging Cell.

[97] Cui W., Xiao H., Wen X., Li C., Bao S., Zeng J., Li Y., Qiao Y., Wang K., Wang H. (2026). Identifying a cancer therapeutic target: Cell-SELEX identifies a membrane protein for aptamer-mediated growth suppression. Proc. Natl. Acad. Sci. USA.

[98] Ferrucci L., Fabbri E. (2018). Inflammageing: Chronic inflammation in ageing, cardiovascular disease, and frailty. Nat. Rev. Cardiol..

[99] Harris T.B., Ferrucci L., Tracy R.P., Corti M.C., Wacholder S., Ettinger W.H., Heimovitz H., Cohen H.J., Wallace R. (1999). Associations of elevated interleukin-6 and C-reactive protein levels with mortality in the elderly. Am. J. Med..

[100] Varadhan R., Yao W., Matteini A., Beamer B.A., Xue Q.L., Yang H., Manwani B., Reiner A., Jenny N., Parekh N. (2014). Simple Biologically Informed Inflammatory Index of Two Serum Cytokines Predicts 10 Year All-Cause Mortality in Older Adults. J. Gerontol. A Biol. Sci. Med. Sci..

[101] Chia R., Moaddel R., Kwan J.Y., Rasheed M., Ruffo P., Landeck N., Reho P., Vasta R., Calvo A., Moglia C. (2025). A plasma proteomics-based candidate biomarker panel predictive of amyotrophic lateral sclerosis. Nat. Med..

[102] Zhang Q., Vallerga C.L., Walker R.M., Lin T., Henders A.K., Montgomery G.W., He J., Fan D., Fowdar J., Kennedy M. (2019). Improved precision of epigenetic clock estimates across tissues and its implication for biological ageing. Genome Med..

[103] Nelson P.G., Promislow D.E.L., Masel J. (2020). Biomarkers for Aging Identified in Cross-sectional Studies Tend to Be Non-causative. J. Gerontol. A Biol. Sci. Med. Sci..

[104] Argentieri M.A., Xiao S., Bennett D., Winchester L., Nevado-Holgado A.J., Ghose U., Albukhari A., Yao P., Mazidi M., Lv J. (2024). Proteomic aging clock predicts mortality and risk of common age-related diseases in diverse populations. Nat. Med..

[105] Donmez T.B., Mansour M. (2026). From prediction to mechanism: Explainable AI uncovers plasma and CSF proteomic signatures of Alzheimer’s disease. J. Proteom..

[106] Lee J.K., Bettencourt R., Brenner D., Le T.A., Barrett-Connor E., Loomba R. (2012). Association between serum interleukin-6 concentrations and mortality in older adults: The Rancho Bernardo study. PLoS ONE.

[107] Assunção L.G., Eloi-Santos S.M., Peixoto S.V. (2012). High sensitivity C-reactive protein distribution in the elderly: The Bambuí Cohort Study, Brazil. Braz. J. Med. Biol. Res..

[108] Chen J., Zhao X., Zhang W., Zhang T., Wu S., Shao J., Shi F.D. (2023). Reference intervals for plasma amyloid-β, total tau, and phosphorylated tau181 in healthy elderly Chinese individuals without cognitive impairment. Alzheimer’s Res. Ther..

[109] Ashton N.J., Pascoal T.A., Karikari T.K., Benedet A.L., Lantero-Rodriguez J., Brinkmalm G., Snellman A., Schöll M., Troakes C., Hye A. (2021). Plasma p-tau231: A new biomarker for incipient Alzheimer’s disease pathology. Acta Neuropathol..

[110] Mehta T., Buzkova P., Kizer J.R., Djousse L., Chonchol M., Mukamal K.J., Shlipak M., Ix J.H., Jalal D. (2017). Higher plasma transforming growth factor (TGF)-β is associated with kidney disease in older community dwelling adults. BMC Nephrol..

[111] Arroyo-Currás N., Somerson J., Vieira P.A., Ploense K.L., Kippin T.E., Plaxco K.W. (2017). Real-time measurement of small molecules directly in awake, ambulatory animals. Proc. Natl. Acad. Sci. USA.

[112] Downs A.M., Plaxco K.W. (2022). Real-Time, In Vivo Molecular Monitoring Using Electrochemical Aptamer Based Sensors: Opportunities and Challenges. ACS Sens..

[113] Wang B., Zhao C., Wang Z., Yang K.A., Cheng X., Liu W., Yu W., Lin S., Zhao Y., Cheung K.M. (2022). Wearable aptamer-field-effect transistor sensing system for noninvasive cortisol monitoring. Sci. Adv..

[114] Wang L., Li J., Zhao Z., Xia Y., Xie Y., Hong D., Liu Y., Tan W. (2024). Aptamer Conjugate-Based Ratiometric Fluorescent Probe for Precise Imaging of Therapy-Induced Cancer Senescence. Anal. Chem..

[115] Kirsher D.Y., Chand S., Phong A., Nguyen B., Szoke B.G., Ahadi S. (2025). Current landscape of plasma proteomics from technical innovations to biological insights and biomarker discovery. Commun. Chem..

[116] Wang B., Pozarickij A., Mazidi M., Wright N., Yao P., Said S., Iona A., Kartsonaki C., Fry H., Lin K. (2025). Comparative studies of 2168 plasma proteins measured by two affinity-based platforms in 4000 Chinese adults. Nat. Commun..

[117] Joshi A., Mayr M. (2018). In Aptamers They Trust: The Caveats of the SOMAscan Biomarker Discovery Platform from SomaLogic. Circulation.

[118] Maretty L., Gill D., Simonsen L., Soh K., Zagkos L., Galanakis M., Sibbesen J., Iglesias M.T., Secher A., Valkenborg D. (2025). Proteomic changes upon treatment with semaglutide in individuals with obesity. Nat. Med..

[119] Jankowski W., Lagassé H.A.D., Chang W.C., McGill J., Jankowska K.I., Gelinas A.D., Janjic N., Sauna Z.E. (2020). Modified aptamers as reagents to characterize recombinant human erythropoietin products. Sci. Rep..

[120] Kuliesius J., Badonyi M., Navarro P., Marsh J.A., Klaric L., Wilson J.F. (2025). Epitope Effect Prevalence in Affinity-based pQTL studies. bioRxiv.

[121] Panikkar D., Vivek S., Crimmins E., Faul J., Langa K.M., Thyagarajan B. (2023). Pre-Analytical Variables Influencing Stability of Blood-Based Biomarkers of Neuropathology. J. Alzheimer’s Dis..

[122] Moqri M., Herzog C.M.S., Goeminne L.J.E., Poganik J.R., Barzilai N., Belsky D.W., Betts-LaCroix J., Chen B.H., Chen M., Cohen A.A. (2024). Challenges and recommendations for the translation of biomarkers of aging. Nat. Aging.

[123] Xu L., Jiang B., Qiu W., Jiang C., Zhang F., Tan W. (2024). The application of aptamers in the field of aging and age-related diseases. Clin. Transl. Discov..

[124] Jaffe G.J., Westby K., Csaky K.G., Monés J., Pearlman J.A., Patel S.S., Joondeph B.C., Randolph J., Masonson H., Rezaei K.A. (2021). C5 Inhibitor Avacincaptad Pegol for Geographic Atrophy Due to Age-Related Macular Degeneration: A Randomized Pivotal Phase 2/3 Trial. Ophthalmology.

[125] Khanani A.M., Patel S.S., Staurenghi G., Tadayoni R., Danzig C.J., Eichenbaum D.A., Hsu J., Wykoff C.C., Heier J.S., Lally D.R. (2023). Efficacy and safety of avacincaptad pegol in patients with geographic atrophy (GATHER2): 12-month results from a randomised, double-masked, phase 3 trial. Lancet.

[126] Hernández-Jiménez M., Martín-Vílchez S., Ochoa D., Mejía-Abril G., Román M., Camargo-Mamani P., Luquero-Bueno S., Jilma B., Moro M.A., Fernández G. (2022). First-in-human phase I clinical trial of a TLR4-binding DNA aptamer, ApTOLL: Safety and pharmacokinetics in healthy volunteers. Mol. Ther. Nucleic Acids.

[127] Hernández-Jiménez M., Abad-Santos F., Cotgreave I., Gallego J., Jilma B., Flores A., Jovin T.G., Vivancos J., Hernández-Pérez M., Molina C.A. (2023). Safety and Efficacy of ApTOLL in Patients With Ischemic Stroke Undergoing Endovascular Treatment: A Phase 1/2 Randomized Clinical Trial. JAMA Neurol..

[128] Wang L., Yu Y., Ni S., Li D., Liu J., Xie D., Chu H.Y., Ren Q., Zhong C., Zhang N. (2022). Therapeutic aptamer targeting sclerostin loop3 for promoting bone formation without increasing cardiovascular risk in osteogenesis imperfecta mice. Theranostics.

[129] Zhang H., Yu S., Ni S., Gubu A., Ma Y., Zhang Y., Li H., Wang Y., Wang L., Zhang Z. (2023). A bimolecular modification strategy for developing long-lasting bone anabolic aptamer. Mol. Ther. Nucleic Acids.

